# Supplementary material for: Enhancing Perspective in Global Health: A Case Study on an International Ophthalmology Partnership
Source: MedEdPORTAL. 2024 Aug 9;20:11431. doi: 10.15766/mep_2374-8265.11431 (PMC11310288; doi:10.15766/mep_2374-8265.11431)
Supplement: Supplementary file 1 — Case Study - Facilitator Version.docxCase Study - Learner Version.docxSession Evaluation Questionnaire.docx [file mep_2374-8265.11431-s001.zip › B. Case Study - Learner Version.docx]

**Enhancing Perspective in Global Health**

A Case Study on an International Ophthalmology Partnership

**Authors:** Michael C. Chen, MD^1,2^, Judy Ndiritu, MBBS^3^, Subash Bhatta, MD^4^

^1^ Associate Professor, Department of Ophthalmology, University of Colorado School of Medicine, Colorado, USA

^2^ Ophthalmology Division Chief, Denver Health Medical Center, Colorado, USA

^3^ Consultant Ophthalmologist, Vitreoretinal and Uveitis Specialist, Nyeri County Referral Hospital, Nyeri, Kenya

^4^ Teaching Ophthalmologist, Vitreoretinal Consultant, Pacific Eye Institute, Suva, Fiji

**Educational Objectives:**

1. Identify possible motivations of various individuals and institutions and explore what each party could potentially offer or gain in an international partnership scenario
2. Describe how disparities in capital (i.e. funds, resources, equipment, networks, training opportunities, etc.) can influence the power dynamics of a partnership
3. Discuss how communication is affected by power and cultural dynamics, identify possible communication gaps within international partnerships, and discuss ways to eliminate these gaps in order to prevent misunderstandings and minimize assumptions
4. Describe the characteristics of healthy and unhealthy partnerships, and the potential benefits and costs of international partnerships
5. Identify cross-cultural issues and discuss the importance of cross-cultural awareness and humility in international partnerships
6. Compare the similarities and differences in principles and themes of practicing ophthalmology both internationally and domestically

**Disclaimer:**

All names, characters, locations, and events portrayed in this case study are fictitious. Any resemblance to actual events, locations, or persons is entirely coincidental.

This page is intentionally blank.

1. **Sarah**

Sarah is a first year resident at an ophthalmology training program at America East University (AEU) in the United States. She became intrigued by the field of ophthalmology during medical school when she first witnessed a cataract surgery on her clinical rotations and saw what a significant impact this short surgery had on the patient when the patch was removed the next day. What ultimately confirmed her choice of the field, though, was when as a medical student she listened to a talk given by Dr. Smith, an ophthalmology faculty at AEU, about the vast number of unnecessarily blind people in the developing world. As she found, the majority of this blindness was due to cataracts, and patients could have this addressed in a matter of minutes for less than $50. As she was pleased to find out from Dr. Smith, a number of organizations exist that enable American ophthalmologists to volunteer a few weeks a year going overseas to perform surgery for the poor. Having developed a heart for the poor as well as a sense of adventure in college since a life-transforming global brigade trip to a country in Central America, she thought that ophthalmology would be a perfect fit to combine her career with her other interests.

Ever since hearing Dr. Smith’s talk and then learning about the partnership that the ophthalmology department at AEU had with Eastern Africa University (EAU), Sarah was hoping that she could stay at AEU for her ophthalmology training. Sarah had never been to Africa, and she was hoping to take advantage of the opportunities that the residents had to travel abroad. Sarah was so excited when she opened the match results and found out that in fact she was going to stay at AEU for her ophthalmology residency.

Sarah had always been a planner and forward thinker, and even before residency had started, had reached out to Dr. Smith and secured the opportunity to join Dr. Smith on his next 2-week trip to EAU in the Fall. By then, Sarah would have only been a few months into her residency, though given the number of blind people and the shortage of eye care providers in the developing world that she had read about, Sarah was eager to help in whatever way she could.

Fall arrived, and everything with the trip logistics were going as planned for Sarah, except that 3 days before the date of departure Dr. Smith emailed her apologizing that something had unexpectedly come up and that he would need to push his departure date back a week. Dr. Smith gave Sarah the option to push her departure date back also, or to go ahead as planned and for Dr. Smith to meet up with her a week later. Dr. Smith mentioned that Dr. Enzi, the local partner at EAU, was there, and should be able to get her oriented and supervise her during this first week. Sarah didn’t know much about Dr. Enzi, and the thought of arriving there alone without Dr. Smith worried her. But after going back and forth and thinking about all the logistics she had already arranged with her residency program and the money she had already put into the trip, and not wanting to shorten her trip in half, she decided to go ahead and start the trip alone.

About 26 hours after Sarah left her apartment, she was relieved to have finally arrived at the hospital grounds at EAU. It was mid-morning. Many unfamiliar sights, sounds, and smells caught Sarah’s attention, but something that really struck her was the crowd of people in the hospital courtyard waiting to be seen. The staff seemed a little confused by Sarah on her arrival, and after she mentioned Dr. Smith, the staff asked her to wait. After about 15 minutes, Manu, an ophthalmology resident at EAU, came out to greet Sarah, apologizing that Dr. Enzi was busy in a meeting. Manu offered Sarah the opportunity to take the day to rest, which Sarah declined, as she was excited to jump into what she had set out to do.

While Sarah felt like a fish out of water upon arrival, over the next week she felt she was acclimating well. While she spent most of her time following Manu seeing patients in the clinic and watching Manu perform a few surgeries in the operating theater, Sarah was pleased to spend some time each morning delivering lectures to the residents and the eye staff. Sarah was a little disappointed that she had minimal interaction with Dr. Enzi, yet when she came to learn that he was the head of the department, she understood why Dr. Enzi was always running in between his numerous duties. Toward the end of the week, Sarah was able to briefly catch Dr. Enzi in the hallway on the way to a meeting, and thanked him for the opportunity to visit his hospital and asked him for the opportunity to perform a cataract surgery. Dr. Enzi smiled and told Sarah to arrange this with Manu.

Sarah was very grateful for Manu, who instructed her on the steps of manual small incision cataract surgery (MSICS) while looking over her shoulder. Sarah was also very grateful that the patient did not speak English and did not appear to understand her need for directions from Manu during the surgery. The case took almost 2 hours and she went through 5 vials of viscoelastic, but Sarah was grateful that the theater staff seemed gracious about this as they were always smiling. Despite all of this, overall she felt that the case went well as she did not rupture the posterior capsule and was able to get in the intraocular lens, and she was so inspired when the patch came off the next day and the patient stood up singing and dancing and showering her with blessings.

As the weekend was approaching, Sarah was excited in making arrangements to visit the nearby wildlife park that she had researched out on tourist websites prior to her departure. Thinking that this would be a good opportunity to thank Manu as well as to have a local companion with her, she invited Manu to go with her and offered to pay for the costs associated with the trip. Sarah was disappointed when Manu turned down the invitation, as he had to spend the weekend studying for a residency exam.

Although Sarah felt like she had acclimated well after her first week, there were still some things that she felt she did not completely understand and which she felt uncomfortable. On the 8^th^ day, Sarah was delighted and somewhat relieved to see Dr. Smith arrive. Shortly after Dr. Smith’s arrival, she watched as Dr. Smith operated from the table that Dr. Enzi usually operates from. Sarah felt fulfilled at the impact that Dr. Smith and she had in helping out the patients, Dr. Enzi, and the ophthalmology department at EAU.

On the plane trip back with Dr. Smith, in reflecting on her time at EAU and the entire experience, Sarah told Dr. Smith that this trip confirmed her desire to continue to participate in global ophthalmology throughout her training and career.

**Part I: Discussion Questions**

1. **Motivations and Roles:** What may be some of Sarah’s motivations and intentions in wanting to travel to Eastern Africa as a first year resident? What may Sarah be perceiving as her role on her trip to EAU? What type of educational and patient care activities may be appropriate for Sarah to participate in during this trip, and why? As Sarah is a first year resident, in what ways may this be the same, or different, if she were a senior resident, or a fully trained attending?
2. **Providing benefit:** Sarah felt fulfilled by the impact that she and Dr. Smith had on those at EAU. How could this impact be quantified? In what ways may Sarah’s visit have benefitted those at EAU, whether it be Dr. Enzi, the residents, the staff, or the patients?
3. **Receiving benefit:** In what ways may Sarah have benefitted through her visit to EAU?
4. **Attitudes and Approach:** Discuss ways in which Sarah approached the trip with an attitude that may have unintentionally communicated a message of implicit condescension.
5. **Ethics:** Discuss whether or not the patient who Sarah operated on would have agreed to the surgery had the patient been informed that Sarah was a trainee with significantly less surgical experience than the other local providers at the hospital. Was the patient in a position to clarify and request who was involved in her care? In a situation where there may be legal/ethical/cultural constraints regarding Sarah performing surgery, who may be responsible that Sarah be made aware of these issues?
6. **Preparation:** What could Sarah have done to be better prepared for the trip? What could she have done to get a better understanding of what to expect as well as what was expected of her?
7. **Narratives and perceptions:** How might Sarah’s perception of those at EAU have been influenced by how Dr. Smith talked about those at EAU? How might her perception about the “developing world” be influenced by what she has heard through popular media or through academic literature? How might the commonly used terms such as “developing world” and “poor” impact her perceptions of the people and place she is visiting? What are other possible ways Sarah might learn about EAU and how might this influence her perceptions differently?

This page is intentionally blank.

1. **Dr. Smith**

Dr. Smith was very excited that Sarah had reached out to him to accompany him on his second trip to Eastern Africa University (EAU). About a decade ago he was in similar shoes, when as a 2^nd^ year resident he took his first overseas trip to an institution in West Africa. The experience was life-transforming, and confirmed his resolve to dedicate a significant portion of his career to global ophthalmology. Throughout the remainder of residency and fellowship, Dr. Smith sought out various opportunities and had traveled to 4 additional sites in 3 developing countries. After completing his training, Dr. Smith took the initiative to take a one-week wet lab course to learn manual small incision cataract surgery (MSICS) hosted by American ophthalmologists with overseas experience, as there were no faculty during his training who he could learn this from. Within his first two years of practice, Dr. Smith traveled to 4 different countries for one to two weeks at a time to partner with local health care providers and perform cataract surgery for the poor, and became the youngest ophthalmologist to be awarded the humanitarian award by his local ophthalmology chapter.

Four years ago (shortly after receiving this award), Dr. Smith received an email from the chair of the ophthalmology department at America East University (AEU), the leading academic institution in the region. The chair had expressed a desire to start a global ophthalmology program. The department was already well-respected nationally, but the chair had the vision to extend the reach of the department on an international level. The department had just secured a generous endowment from a Western non-profit organization, Global Vision Savers (GVS), who focused on addressing blindness in Africa, and recently completed a fundraising campaign for children’s blindness. The chair was recruiting a faculty member to head the global effort for the department. To attract Dr. Smith, he was offered the title of “Director of Global Programs” from the start. Given Dr. Smith’s interests, this was a position that he could not turn down, and he readily accepted.

In looking for international partnerships, Dr. Smith was pleased to find out that GVS already had a contact with the ophthalmology department at EAU, and based on what he learned from the board members at GVS, they could really benefit from Dr. Smith’s help. Three years ago, at the annual American Academy of Ophthalmology meeting, a representative from GVS introduced Dr. Smith to Dr. Enzi, the head of the ophthalmology department at EAU. After talking with Dr. Enzi for about half an hour, Dr. Smith convinced Dr. Enzi to postpone his return flight home and to reroute his flight itinerary to pay a short visit to the ophthalmology department at AEU.

Dr. Smith’s first trip to EAU was a few months after this, when he went to visit for 2 weeks at Dr. Enzi’s invitation. Dr. Smith was very impressed by the reception he was given. It seemed that everybody at the institution was so eager to learn and hear what he had to say. They had even asked him to help with the surgery backlog. Dr. Smith felt awkward admitting it, but he was surprised to see how adept Dr. Enzi was at MSICS. On a case that would take Dr. Smith one hour to do, Dr. Enzi was able to do in less than 10 minutes. Dr. Smith felt awkward when some patients would noticeably express their desire for him rather than Dr. Enzi to perform their surgeries. This happened several times that week, and each time it happened, Dr. Smith looked over to Dr. Enzi, who would smile and nod. Dr. Smith, out of politeness, obliged.

On return from that trip, Dr. Smith was pleased to report to his chair that a partnership had been solidified. Dr. Smith and his chair were excited about all the potential opportunities, not only the opportunities to help the poor patients and the providers at EAU, but also the immersive opportunities for their American residents. On top of this, this partnership seemed to open doors for many potential research projects. The chair communicated these opportunities to GVS, who shared in the excitement and vowed continued support and collaboration for these endeavors.

Since Dr. Smith’s first visit, 3 residents from his program had had the opportunity to visit EAU individually as part of their international elective. All 3 came back with glowing reviews of their experience. One aspect that consistently elicited much positive feedback was the ability to learn and perform MSICS. The returning residents brought back numerous stories and photos, which were shared with the AEU development office and featured in the bi-annual department newsletter.

Dr. Smith couldn’t believe how fast time had flown and that it had already been 2 years since the first trip. This upcoming trip was Dr. Smith’s second trip to EAU. Due to the increasing workload of his other institutional responsibilities, Dr. Smith did not have the time to travel as much as he would like, but he understood the importance of making periodic visits to maintain the partnership. It worked out well that he could be there with Sarah, given that Sarah was a beginning resident and was traveling at an earlier stage of training than the other residents.

Unexpectedly, 3 days before the date of departure, his chair called him and informed him of a VIP patient who was flying into town next week for her eye appointment. The chair wanted Dr. Smith to join them for dinner the evening after the appointment. Dr. Smith informed the chair of his upcoming trip, but the chair convinced him to push his departure date back, as this patient was a potential donor for AEU’s long dreamed-of new eye building. As the chair explained, on previous visits, this patient seemed intrigued about the department’s international work, and as the development office had advised, a brief presentation from Dr. Smith over dinner could possibly be the key that would unlock the donor pledge. Dr. Smith was torn, but ultimately gave in to his chair’s request. Informing Sarah that something had come up and that his departure would have to be postponed, Dr. Smith convinced himself that Sarah would be okay going ahead of him, as Dr. Enzi and his team seemed so hospitable and accommodating on his first visit.

Dr. Smith was relieved when upon his arrival it seemed like Sarah was having a good time. Shortly after arriving, Dr. Smith was asked by Dr. Enzi if he could help with the surgeries. Dr. Smith had not intended to operate on this short trip, particularly after seeing on his previous trip how much better Dr. Enzi and the other faculty were at MSICS than he was. But Dr. Enzi seemed insistent that his help was needed, and out of courtesy, Dr. Smith gave in to the request.

While in the operating theater, Dr. Smith noticed something that wasn’t there on his first trip: an older-appearing phacoemulsification machine in the corner. Having not done MSICS in over a year and after struggling through a few cases to get oriented again, Dr. Smith was relieved when Dr. Enzi asked him to supervise him on the phacoemulsification machine. While phacoemulsification was definitely something Dr. Smith felt more comfortable doing, unfortunately things were still a struggle. The machine—a generation older than the one Dr. Smith trained on himself while in residency—definitely did not perform as well as the machine that he was now operating with back in the US. But Dr. Smith made an intentional effort to keep silent about the nicer machine he was operating with in the US. Midway through supervising Dr. Enzi on the case, Dr. Smith came to realize that it wasn’t just Dr. Enzi’s inexperience with phacoemulsification or the older technology of the machine, but that the vacuum mechanism was not functioning properly. After he watched Dr. Enzi finish the case with some ingenuity, they decided to abandon the use of the machine for the remaining cases and to resume MSICS.

**Part II: Discussion Questions**

1. **Motivations**: What may be the motivations of the parties introduced in this section—Dr. Smith, the ophthalmology chair at AEU, the non-profit GVS—in the partnership with the ophthalmology department at EAU? In the situation described, to who are these parties (i.e. Dr. Smith, the chair, GVS) accountable to? Assuming they are accountable to multiple parties, how should each of these parties prioritize the order of accountability?
2. **Benefits**: How does or how can Dr. Smith/AEU or GVS add value to this partnership that would benefit those at EAU?
3. **Burdens:** In what ways might those at EAU be burdened by Sarah’s and Dr. Smith’s visit?
4. **Power and approach:** In the situation described, discuss who may be driving the partnership. What are the implications for the partnership when the invitation for help and other communication comes through a third party like GVS?
5. **Ethics:** In what ways can the local patients potentially benefit from the partnership? In what ways can the local patients potentially be harmed by the partnership? Do the local patients have any chance of getting worse treatment with the presence of visiting faculty and residents?
6. **Ethics:** What do you think would be an appropriate approach towards potential research projects through this partnership?
7. **Narratives and perceptions:** What are some preconceived notions Dr. Smith may have had for him to be surprised by how good Dr. Enzi was at MSICS? Discuss possible reasons why patients would request Dr. Smith rather than Dr. Enzi to perform their surgeries. How should Dr. Smith and Dr. Enzi have each responded in these situations?
8. **Narratives and perceptions:** Why might Dr. Enzi have asked for Dr. Smith to help with the surgical backlog? If you were Dr. Smith and understood what these reasons were, would you still operate?
9. **Preparation:** If you were Dr. Smith, how would you go about advising yourself, as well as your residents who visit EAU, in pre-trip preparation and post-trip debrief to uphold the dignity and respect of those at EAU? Should there be a selection process of which residents are allowed to visit, and if so, how would you go about structuring this process?

This page is intentionally blank.

1. **Manu**

Manu, a senior ophthalmology resident at Eastern Africa University (EAU), remembers growing up wanting to be a doctor after seeing how much joy was brought to his extended family when his older cousin scored well on his university entrance exams and secured a position at the country’s top medical school. When it came Manu’s time to take this exam, he did not do as well as his cousin, but through support from American donors who he knew through projects they had supported in his home town when he was growing up, he was able to obtain his medical degree through a new private medical school in his region. During medical school, he learned about the number of people blind from glaucoma, and was grateful to have secured a position in the new ophthalmology residency program that was starting at EAU.

Shortly after starting residency, he was intrigued by this partnership that EAU started with an institution in the United States (US). Having heard about the US through the various American volunteers that had visited his hometown growing up, he was hoping to one day visit the US to see for himself what America was like. Manu had heard from Dr. Enzi that through this partnership that there may be opportunity at some point for the residents to visit the American institution and that they were working on the arrangements. Hearing that only Dr. Enzi had visited the US once, and that the other faculty members had not visited yet, he wasn’t sure if or when this day would come.

With the previous 3 American residents, Manu didn’t have much interaction with them, as they were with Dr. Enzi most of the time. It was a curiosity to Manu that Dr. Enzi, being the head of the department and as important and as busy as he was, was spending so much time attending to the American residents during their visits. When Dr. Enzi’s secretary had unexpectedly asked him to attend to Sarah when she arrived, Manu didn’t know why it was different this time, though he didn’t question it.

The usual routine for the residents and the staff was different the first week that Sarah was there for several reasons, one being that she volunteered to give some lectures, and to accommodate her request they carved out time in the mornings for her to do this. Manu found Sarah’s lectures on phacoemulsification interesting with nice videos and photos, though he didn’t know how this applied to his own training. He was surprised to learn after one of the lectures that Sarah had just started her residency, given how confident she was in presenting the videos. On further inquiry, Manu came to learn that the videos Sarah was showing were not of herself but of her professors operating.

Most of the surgery cases that occurred at EAU were performed by Dr. Enzi and the 4 other faculty members. As a resident, Manu only had a select number of cases assigned to him, as most of his cases he did were done offsite at outreach camps. When Manu heard from Sarah that Dr. Enzi had told her to ask him about performing a cataract surgery, even though he was not told the reason why, and even though this meant one less case for him, he knew that his role was to follow orders. It was rough walking Sarah through the steps of MSICS, as Manu was in the process of learning himself. They used up the last vials of viscoelastic, and the remaining 3 cases that day had to be put on hold. Fortunately, the next day additional supplies arrived and cases could continue.

Despite the extra work it was for Manu to have Sarah around, she seemed appreciative of his efforts to make her feel welcomed. Manu felt torn when she asked him to accompany her over the weekend to the wildlife park. Manu had always wanted to visit the wildlife park ever since he was a little kid, as he heard about a park where mostly Western tourists went to visit. It was not within the means of his family to visit, and at his monthly stipend as a resident, it certainly was not within his means to visit now. It was difficult to fathom, given the cost of admission to the park, how Sarah as a resident like him was able to not only go herself but even offer to pay for him. It was a generous offer but something he could not accept. Having grown up in a conservative mixed Christian/Muslim society, he did not know how it would be viewed if he were to travel over the weekend with a female, let alone a female who dressed in a way considered indecent by his elders. It was difficult to have to turn down this opportunity, though in the end he did so, using the excuse that he had to study for his exams.

Manu saw much less of Sarah over the next week once Dr. Smith arrived. At the end of Sarah’s visit, they exchanged emails. Perhaps one day Sarah would be a connection for him to visit the US and perhaps she would be someone who would open the doors for him to pursue additional training opportunities.

**Part III: Discussion Questions**

1. **Costs/Benefits:** In contrast to the characters of the previous two narratives, Manu had no say in the formation or participation in this partnership. Discuss the ways in which Manu may have been better off with or without this partnership. How could this partnership have been structured to create a balance between providing Sarah some valuable learning opportunities while also maintaining the best interests of the local patients and local residents like Manu?
2. **Narratives and Perceptions:** How could the support of American donors that Manu received when growing up as well as for his education influence Manu’s perception of the US, and how could this affect his relationships with Americans going forward?
3. **Disparity:** In a situation where an American resident like Sarah may have more material wealth than many of her hosts, how should Sarah navigate the fine line between generosity and paternalism?
4. **Power and Approach:** What may be the reasons Dr. Enzi appeared to be more attentive to the American residents than to his own residents? How might different cultural teaching approaches affect the attending/resident relationship and how might this lead to miscommunication in an international partnership? How might Manu be influenced and shaped by observing Dr. Enzi’s interactions with the Americans?
5. **Costs/Benefits:** Reflect on the benefit and costs of Sarah’s lectures to those at EAU. How could Sarah be involved in classroom learning that would be more relevant and beneficial to all parties involved? With Sarah, as a beginning resident, providing lectures to those at EAU, what implicit messages may have been communicated to the residents and attendings at EAU about their own education and training?
6. **Reciprocity:** How might Dr. Enzi’s and Manu’s hospitality and attentiveness be reciprocated by Dr. Smith, Sarah, and those at AEU? If Manu were to ever get the chance to visit the US as a resident or an attending, in what ways would this experience benefit him? What are things that he may learn that would be applicable to his practice in his home country?

This page is intentionally blank.

1. **Dr. Enzi**

Dr. Enzi remembers as a child watching his grandmother go blind as her eyes became white, which now looking back he thinks most likely were cataracts. He remembers how his younger brother stayed home to take care of his grandmother while he went to school. He remembers how a few years after his grandmother passed away he watched a team of European doctors come visit his town and witnessed blind people regaining their sight through what he learned was cataract surgery. He dreamt of one day being that kind of doctor for his own people, and through much hard work and a series of fortunate events, including a sponsorship by a Western non-governmental organization, he was able to secure ophthalmology residency training in a neighboring country, as there were none in his own country at that time. Immediately after finishing his training, he was posted to a hospital in a remote town where he was the only ophthalmologist for a region of over 1 million people.

Nine years later, due to several factors, one of the major ones being his conviction to further the training opportunities within his own country, he relocated to a larger city to work at Eastern Africa University (EAU), which had recently started the second ophthalmology training program in the country. His medical and administrative acumen was quickly recognized, and within the first year he was promoted to the head of the department. Not only did this new position fit his gifts and abilities well, but the relocation was better for his family. His wife was happier in a larger city, and there were better schools and opportunities for his two children. In his department, Dr. Enzi was no longer alone. He now had 4 other ophthalmology colleagues in his department, but the need for more eye care providers and more infrastructure was impossible to ignore. He cherished his role as the head of the department, hoping that through the patient care and education efforts of his department that he could influence the eye care situation in his region and in his country.

Dr. Enzi was very grateful when 3 years later, at a regional ophthalmology meeting, he was introduced to an American who represented the non-governmental organization Global Vision Savers (GVS). GVS offered their help to strengthen the work of his department to improve the eye care situation in his region, and when an agreement was met, Dr. Enzi caught the glimpse of hope that some of the challenges that he encountered on a day-to-day basis would be surmountable. Prior to this partnership, Dr. Enzi faced numerous challenges, including wrestling with authorities to procure basic equipment, instruments, and consumable supplies to care for his patients. At times, months of waiting would conclude with the items not arriving for various reasons, or arriving after they were long-expired. At other times, when the items were available, the associated fees were so exorbitantly expensive that it was not financially feasible to obtain them. From the start, GVS provided his department with a better operating microscope, 10 instrument trays, and with some regularity would send supplies of consumables, such as intraocular lenses and viscoelastics. GVS seemed particularly interested in the eye health of children and in pediatric cataracts, which though Dr. Enzi encountered this condition from time to time, did not present to his facility with the frequency that was reported in other regions in the country. Regardless, the supplies were put to good use, where the surgery volume, which consisted mostly of adult cataracts, rose by over 200% in the first year, largely in part through GVS’s support.

Dr. Enzi has fond memories of his trip to the Unites States (US) 3 years ago when he was sponsored by GVS to go to the American Academy of Ophthalmology (AAO) meeting. Along with the wealth of knowledge being shared at the meeting, what Dr. Enzi still vividly remembers was the large exhibition hall with all the vendors exhibiting their products and services. Dr. Enzi couldn’t quite get around the stark contrast of what he encountered on an everyday basis with what he observed about the vast amount of wealth that was tied to eye care in other parts of the world. He was impressed by the phacoemulsification machines and the beautiful surgical videos being presented by the speakers. Although he wasn’t sure how or when this technology could be sustainable in his current system and patient population, he dreamt of learning the technique in hopes of one day being able to offer this service to his patients.

It was at this meeting that Dr. Enzi was introduced to Dr. Smith from America East University (AEU). Dr. Enzi had been hoping for a partnership with an academic institution in the US, as the other training program in his country had already entered such a partnership. Formalities were exchanged during that initial conversation and Dr. Enzi was not able to remember it all, but he does remember Dr. Smith discussing their shared interest in helping the underserved, and mentioning his interest in MSICS and opportunities for his residents. At the end of the conversation, Dr. Smith invited him to adjust his trip itinerary and to reroute his flights to make a brief visit to AEU. With Dr. Enzi’s numerous responsibilities back at home, the one week trip for the AAO meeting was tough enough to carve out of his schedule, and extending out his trip a few days more was even tougher. But if a partnership with a US institution was what his department needed, he felt that turning down this invitation from a potential partner was not an option.

During the 3 days of Dr. Enzi’s visit to AEU, he spent most of his time observing Dr. Smith examining patients in clinic and performing surgeries in the operating room. As he learned from Dr. Smith, US law prohibited visiting foreign medical providers like himself from handling patients. One thing that Dr. Enzi did not expect to learn during his time at AEU was that despite all the wealth that he had heard about in the US and which he was now observing first hand, that there still appeared to be many problems with their system and that they had their own issues with patient populations who were underserved. These types of patients who came through appeared to frustrate Dr. Smith, and when Dr. Enzi inquired further, Dr. Smith responded that these problems were hopeless to try to solve.

Dr. Enzi was impressed with how nice the facilities and equipment were at AEU, and found it strange that despite the vast space in the clinic and operating room, filled with costly machines that often weren’t being used, that those he met still complained about not having enough space and resources. By the statistics that the chair of the department proudly shared with him regarding the number of patients examined and operated on each year, Dr. Enzi thought to himself about how the number of patients treated through his own department were easily double those numbers, done with significantly less space, less resources, and a smaller number of providers. He wondered what his own department could do if they only had the type of resources that a department like AEU had. And even so, there would still be so much need.

Dr. Enzi was happy when Dr. Smith agreed to visit EAU a few months later. During the 2 weeks of Dr. Smith’s visit, Dr. Enzi made the arrangements to ensure that Dr. Smith had the best experience possible. He reserved his best staff to attend to Dr. Smith. He made sure to set aside a number of surgeries for Dr. Smith to do, made sure to let Dr. Smith use the table and microscope in the operating theater that he normally operated from, and reserved the two best instrument sets for him to use. The usual flow of the clinic and operating theater was put on hold those 2 weeks, and the surgery backlog increased, though Dr. Enzi thought the potential future improvements of eye care as a result of this partnership was worth these inconveniences, if in fact the partnership could be solidified. Dr. Enzi was both relieved and elated when Dr. Smith mentioned at the end of the visit that he wanted to officially establish the partnership.

At the time that Sarah arrived, Dr. Enzi was on an overseas phone call with the board at GVS, reporting statistical outcomes of pediatric cataract surgeries done through his department the last month. Dr. Enzi could sense they were disappointed by the low volume of pediatric cataract surgeries, and he preemptively offered his hypotheses why the volume was not as high as the other centers in the country that GVS was also partnering with. It was a stressful call, as he was afraid that if his department could not produce the numbers that GVS was requesting that they would pull their support.

Dr. Enzi felt ashamed that he was not able to go and greet Sarah when she arrived. Two years ago, when the first American resident from AEU visited, Dr. Enzi wanted to make sure the visiting resident had the best experience possible, and was able to spend a significant time supervising him. But with the growth of his department and his increasing responsibilities, this became increasingly difficult with each following resident. Dr. Enzi was relieved to find out that this time Dr. Smith was going to accompany Sarah for the entire duration and that his supervisory duties would be relieved somewhat. Dr. Enzi was hoping for the opportunity during Dr. Smith’s upcoming visit to discuss with him the increasing challenges of supervising the American residents during their visits, and hoping to use this conversation as a bridge to broach the topic of things that AEU could potentially help EAU with, such as assistance with equipment and supplies and opportunities for members of his department to visit the ophthalmology department at AEU. The airfare and associated costs to go to the US were prohibitive for those in Dr. Enzi’s department, but he didn’t know how to appropriately communicate this. When Dr. Enzi hinted at this barrier to Dr. Smith over email last year, the response from Dr. Smith was that he understood, but unfortunately the funds at AEU were limited and Dr. Smith suggested inquiring with GVS for funding support. Given the circumstances with GVS, Dr. Enzi didn’t think he could make this request of GVS, and thought that perhaps an opportunity would arise for him to talk with Dr. Smith in person if there were any alternative options.

Dr. Enzi was disappointed when he received an email from Dr. Smith a couple weeks ago about how Sarah would be coming alone for the first week, for reasons that were not given. When he was told in the middle of the phone call that Sarah had arrived, he instructed his secretary to ask Manu to attend to her.

A week later, when Dr. Smith arrived, even though it appeared that he was not as excited to operate as he had been on his first visit 2 years ago, Dr. Enzi asked him to do so, as more patients had been coming to the department over the last week because when people saw Sarah, word started to get out that the visiting doctors had arrived. Many patients were requesting that the visiting doctors do their surgeries, and Dr. Enzi wanted to cater to the patient requests the best he could, as community reputation was very important to the survival of his department.

When Dr. Enzi saw Dr. Smith struggle through several MSICS surgeries, he thought it best to divert Dr. Smith by asking him to supervise him on some phacoemulsification cases. Six months ago, Dr. Enzi received an unexpected shipment from GVS of a used phacoemulsification machine. He was wondering how GVS had known of his interest in learning phacoemulsification, and when he inquired further he found out that the board allocated this donation to his facility as they thought it would help his department in removing pediatric cataracts. Since the arrival, the machine mostly sat idle in the corner (which was another point of frustration and disappointment for GVS over the phone call). Dr. Enzi had attempted a few cases on his own earlier in the month, but the cases were much longer and rougher than his MSICS cases and he decided it be best that he revert to MSICS. Now that Dr. Smith was here, perhaps Dr. Smith could supervise and instruct him.

On the one patient that Dr. Enzi operated on with Dr. Smith supervising, Dr. Enzi was somewhat relieved yet also disappointed when Dr. Smith told him that the reason he was having difficulty during the case appeared to be an issue with the vacuum pump. Dr. Enzi attempted to contact the company representative, who was the company representative for 5 African countries. He got in contact with the representative the next day. When the representative found out the equipment was a used model originating from a donation from the US, he advised that those in the US should be contacted. On Dr. Enzi’s behalf, Dr. Smith contacted the company representative in the US that he interacts with. The representative informed him that as the machine was 2 generations outdated, no parts were being produced anymore, and as such the company was not able to offer any support.

At the end of Dr. Smith’s and Sarah’s visit, Dr. Enzi found out from Dr. Smith about AEU’s intention of continuing the partnership. Unfortunately, Dr. Enzi did not find the opportunity where he felt comfortable talking with Dr. Smith about the items he found pressing, and this would have to wait for another time. To sustain the partnership, he had his staff dress in traditional attire and arranged a formal farewell ceremony to communicate their respect and appreciation.

**Part IV: Discussion questions**

1. **Motivations**: What may have been some of Dr. Enzi’s motivations and interests when entering this partnership?
2. **Partnership**: Discuss whether or not Dr. Enzi was in a position to negotiate aspects of the partnership for better opportunities for his department. Consider why at some points in the narrative Dr. Enzi was not able to openly communicate his thoughts and desires with his partners.
3. **Providing benefit:** In what ways has Dr. Enzi provided benefit to Dr. Smith and the AEU residents? The international experience at EAU may have been life-transforming for residents like Sarah. Do you think Dr. Enzi is aware of the impact he has in facilitating these life-transforming experiences?
4. **Power and approach:** Discuss ways in which to navigate the fine line of self-benefit and altruism when institutions like AEU partner with institutions like EAU.
5. **Ethics:** Consider how the different rules between the two countries regarding foreign doctors being able to do hands-on patient care poses a challenge to building an equitable partnership in global ophthalmology. What can be done to balance the learning opportunities for residents and faculty in both countries as there are differing legal provisions regarding handling of patients by foreign doctors? If the partner from a high-income country (HIC) pours resources into the partner in a low/middle-income country (LMIC), under what circumstances would it be reasonable for the visiting faculty and residents to have hands-on learning opportunities in MSICS? If in fact it was not legal for foreign visitors to have hands-on patient care/learning opportunities at Dr. Enzi’s institution, should Dr. Enzi nevertheless let the foreign visitors have hands-on learning opportunities at their institution, as this good gesture can bring his department benefits in other areas though this partnership?
6. **Equipment:** In this situation with the donated broken phacoemulsification machine, what may be appropriate responses/actions for Dr. Smith to take? What are potential issues with donated second-hand equipment/medical supplies? Should equipment or supplies be provided if there is no plan for sustainability? Would a needs-assessment be beneficial in the charity/donation process, and if so, what are factors to consider in the design and implementation of such a process?
7. **Disparity**: In light of the material disparity between Dr. Enzi and Dr. Smith’s institutions/countries, discuss how to navigate the financial aspects of this partnership. In what ways would understanding/transparency regarding the disparity in finances and resources be helpful or harmful in this partnership?
8. **Partnership**: Are global health partnerships necessary for eye institutions in LMICs? In HICs? Why or why not? If so, what would be the purposes of such partnerships? How can host institutions/individuals in LMICs benefit from the partnership with institutions from HICs, and vice versa? In what ways may the appeal of “global ophthalmology” be similar between the partners in HICs and LMICs, and in what ways may the appeal be different?
9. **Partnership:** How would you define “partnership”? How would you describe the partnership in this case study? Discuss aspects in which this partnership is healthy or unhealthy. Evaluate the benefits and costs to the parties involved. How could this partnership be structured to be more equitable and reciprocal?
10. **Preparation:** Discuss ways in which the parties in this case study could have gone about gaining a better understanding on the cultural values and etiquettes of the other parties involved.
