# Supplementary material for: Enhancing Perspective in Global Health: A Case Study on an International Ophthalmology Partnership
Source: MedEdPORTAL. 2024 Aug 9;20:11431. doi: 10.15766/mep_2374-8265.11431 (PMC11310288; doi:10.15766/mep_2374-8265.11431)
Supplement: Supplementary file 1 — Case Study - Facilitator Version.docxCase Study - Learner Version.docxSession Evaluation Questionnaire.docx [file mep_2374-8265.11431-s001.zip › C. Session Evaluation Questionnaire.docx]

**Session Evaluation/Questionnaire**

Thank you for participating in the global ophthalmology case study. We value your feedback. Please complete the following questions. This should take you less than 5 minutes to complete.

*Regarding the CONTENT of the session*

1. The content of the session was effective in helping my understanding of the topic of international partnerships.

- Strongly agree
- Agree
- Neutral
- Disagree
- Strongly disagree

1. The content of the session was effective in helping me formulate my views and perspectives on international partnerships.

- Strongly agree
- Agree
- Neutral
- Disagree
- Strongly disagree

1. The content of the session was effective in increasing my curiosity on the topic of international partnerships.

- Strongly agree
- Agree
- Neutral
- Disagree
- Strongly disagree

*Regarding the FORMAT of the session*

1. The format of the session was conducive to achieving the learning objectives.

- Strongly agree
- Agree
- Neutral
- Disagree
- Strongly disagree

*Regarding the session overall*

1. The learning objectives were made clear.

- Strongly agree
- Agree
- Neutral
- Disagree
- Strongly disagree

1. The learning objectives were met.

- Strongly agree
- Agree
- Neutral
- Disagree
- Strongly disagree

1. I found the session worthwhile.

- Strongly agree
- Agree
- Neutral
- Disagree
- Strongly disagree

*Perspectives and Practice (Open Answer)*

1. How has today's session influenced your views and perspectives on global ophthalmology?
2. After today's session, what is one thing you may change in how you practice ophthalmology during residency?
3. After today's session, what is one thing you may change in how you practice ophthalmology in your future career?
