# Supplementary material for: Enhancing Perspective in Global Health: A Case Study on an International Ophthalmology Partnership
Source: MedEdPORTAL. 2024 Aug 9;20:11431. doi: 10.15766/mep_2374-8265.11431 (PMC11310288; doi:10.15766/mep_2374-8265.11431)
Supplement: Supplementary file 1 — Case Study - Facilitator Version.docxCase Study - Learner Version.docxSession Evaluation Questionnaire.docx [file mep_2374-8265.11431-s001.zip › _Educational Summary Report_11431.pdf]

MedEdPORTAL® | The AAMC Journal of Teaching and Learning Resources

Original Publication

Open Access

Enhancing Perspective in Global Health: A Case Study on an International Ophthalmology Partnership

Michael C. Chen, MD*, Judy Ndiritu, MBBS, Subash Bhatta, MD

*Corresponding author: Michael.2.Chen@cuanschutz.edu

Abstract

Introduction: Global ophthalmology opportunities are becoming increasingly popular, and international partnerships are becoming more common among academic training institutions in the United States. There is need for training in the complex relational, motivational, ethical, and logistical issues that may arise in these partnerships. **Methods:** We developed a 3-hour case-based session featuring four characters in a fictitious international ophthalmology partnership scenario. Facilitators used structured questions for each of the four parts to foster interaction and discussion among learners. After the activity, participants completed an evaluation/questionnaire consisting of Likert-scale and open-ended questions. **Results:** A total of 23 ophthalmology residents and seven medical students underwent the activity over four iterations. The activity was well received, with 100% of learners either strongly agreeing (90%) or agreeing (10%) when asked if the session was worthwhile and 100% of learners either strongly agreeing (87%) or agreeing (13%) when asked if the format was conducive to achieving the learning objectives. Answers to questions on how learners would change how they practice ophthalmology in their residency and in their future careers revolved around the following topics: consideration of other perspectives, humility, self- and situational awareness, complexities of partnerships, reciprocity and exchange, importance of communication, and connection of principles between international and domestic medical practice. **Discussion:** While this case study explores an international ophthalmology partnership scenario, the principles and themes presented can be applicable to other fields of medicine, and can be applicable to the practice of medicine both internationally and domestically.

Keywords
International Partnership, Perspectives, Case-Based Learning, Ethics/Bioethics, Global Health, Ophthalmology

Educational Objectives

By the end of this activity, learners will be able to:

1. Identify possible motivations of various individuals and institutions and explore what each party could potentially offer or gain in an international partnership scenario.
2. Describe how disparities in capital (e.g., funds, resources, equipment, networks, training opportunities, etc.) can influence the power dynamics of a partnership.
3. Discuss how communication is affected by power and cultural dynamics, identify possible communication gaps within international partnerships, and discuss ways to eliminate these gaps in order to prevent misunderstandings and minimize assumptions.
4. Describe the characteristics of healthy and unhealthy partnerships and the potential benefits and costs of international partnerships.
5. Identify cross-cultural issues and discuss the importance of cross-cultural awareness and humility in international partnerships.
6. Compare the similarities and differences in principles and themes of practicing ophthalmology both internationally and domestically.

Citation:
Chen MC, Ndiritu J, Bhatta S. Enhancing perspective in global health: a case study on an international ophthalmology partnership. *MedEdPORTAL*. 2024;20:11431.
https://doi.org/10.15766/mep_2374-8265.11431

Introduction

There is a significant amount of interest in global health within undergraduate and graduate medical education in the United States.[1,2] This interest in global health is certainly present within the field of ophthalmology, given the magnitude of preventable and treatable blindness worldwide. A survey of applicants to US ophthalmology residency programs found that nearly all respondents (95.4%) had an interest in participating in an

Copyright © 2024 Chen et al. This is an open-access publication distributed under the terms of the Creative Commons Attribution license.

international experience during their residency,[3] and a survey of US ophthalmology residency programs showed that among the programs that responded, a majority (88.6%) either offered their residents international experiences or supported them in finding their own experiences to go abroad.[4] Associated with this interest are a growing number of global ophthalmology fellowships offering postresidency training that takes place both at the domestic home institution site and at partner institution sites abroad.[5,6]

International partnerships bring potential for good but also potential for harm for the stakeholders involved. Lu and colleagues, in a systematic review of the impact of global health electives on US medical residents, found that four positive themes emerged: “(1) improvement of medical knowledge, physical examination, and procedural skills, (2) improvement in resourcefulness and cost-effectiveness, (3) improvement in cultural and interpersonal competence, and (4) professional and career development.”[7] However, challenging the assumption that these global health experiences are inherently beneficial to all stakeholders involved, Lu and colleagues also performed a systematic review to summarize the perceptions of low- and middle-income country hosts who interact with high-income country visitors during short-term experiences in global health, where four themes emerged: “(1) sociocultural and contextual differences, (2) institutional and programmatic components, (3) impact on host institutions and individuals, and (4) visitor characteristics and conduct.”[8] This disconnect between the benefits to US learners and the perceptions of low- and middle-income country hosts may explain the potential issues encountered in international partnerships, including ethical and legal challenges, power imbalances, miscommunication, and suboptimal utilization of resources. The potential for unintended harm raises the importance of the need for awareness and training in the complex relational, motivational, ethical, and logistical issues that may arise within international partnerships.

With the intent of developing ethical awareness, White and Evert published a four-part case scenario involving an American medical student traveling to Kenya, following participants’ encouragement when it was presented at a global health conference in 2011 to medical faculty, residents, and students from diverse countries and backgrounds.[9] Current *MedEdPORTAL* resources relevant to global health include simulation and case-based modules on ethics,[10,11] health systems,[12] and evaluation and treatment of various medical conditions[13,14] targeted primarily at the undergraduate medical education audience. Our case study is unique and adds to the list of *MedEdPORTAL* resources by inviting the learner into a deeper exploration of the personal, professional, ethical, and logistical aspects of global health through entering into an international ophthalmology partnership narrative. Following a format similar to White and Evert’s case scenario, this international ophthalmology partnership scenario is viewed through the eyes of four characters. This case study aims to bring about more self- and situational awareness of how these partnerships may be perceived differently by different stakeholders. While the case study is primarily targeted at ophthalmology residents and medical students interested in ophthalmology, those not involved in ophthalmology may still find the activity beneficial as the principles and themes presented in it are not exclusive to ophthalmology but may be applicable to other fields or situations in which international partnerships arise.

The case study method is well recognized within education in various disciplines, including medicine.[15] The learner is invited to engage and think critically within the context of plausible real-world scenarios, with the goal of taking the theory learned and applying it to practice.[16] While there is a growing amount of educational material on ethics and responsible practice as they pertains to global ophthalmology,[17] the format as presented here may be more conducive to reflection and long-term retention. As many ophthalmology residents and medical students have had previous international, cross-cultural, and cross-socioeconomic experiences,[3] this case study invites learners to learn from each other through group discussion as they wrestle with the ambiguities, nuances, and complexities of an international ophthalmology partnership scenario.

Methods

The development of this case study was guided by the Kern approach.[18] Steps 1-3 (problem identification and general needs assessment, targeted needs assessment, and goals and objectives) were performed through multiple discussions and communications among the author group regarding issues encountered in international partnerships, learned either through personal experiences, experiences of colleagues, or the literature. In step 4 (education strategy), the author group decided that a case study format would be most conducive to discussion, reflection, and long-term retention. Steps 5 (implementation) and 6 (evaluation and feedback) are described below. We developed the evaluation/questionnaire with consideration of Kirkpatrick’s hierarchy of outcomes, targeting levels 1 (reaction, with focus on learner satisfaction) and 2 (learning, with focus on change in knowledge, attitudes, and skills).[19]

Copyright © 2024 Chen et al. This is an open-access publication distributed under the terms of the Creative Commons Attribution license.

A total of 23 US ophthalmology residents and seven US medical students from the Penn State College of Medicine and the University of Colorado School of Medicine underwent the activity over the course of four iterations (group sizes of six, five, four, and 15 learners per session, respectively). The duration of each session was 3 hours. At the Penn State College of Medicine, the invitation to participate in the activity was extended to interested learners, and attendance was voluntary (three sessions, total of 15 learners). At the University of Colorado School of Medicine, the activity took place during protected time for didactics, and attendance was required for those without surgical responsibilities (one session, total of 15 learners). This four-part case study invited the learners to view a fictitious international ophthalmology partnership scenario from the vantage point of four characters: an ophthalmology resident from the US, an ophthalmology attending from the US, an ophthalmology resident from Eastern Africa, and an ophthalmology attending from Eastern Africa. The sessions were facilitated by one author (Michael C. Chen) utilizing the narratives and discussion questions contained in the facilitator's version of the case study (Appendix A). The participants received the learner version of the case study (Appendix B) at the session, with each part of the case study handed out as the case unfolded.

The session began by distributing and reviewing the objectives, as well as setting the ground rules for the activity. The session then proceeded to the first part of the case study where one or more participants were asked to read the narrative aloud to the group, followed by discussion prompted by the discussion questions. The process repeated for the remaining three parts of the case study. The approximate time for reading each part aloud ranged from 5 to 14 minutes (specified in the facilitator's version). The time dedicated to discussion was approximately 20-30 minutes per part. The facilitator helped lead discussion points throughout the reading of each part of the case study, with particular emphasis placed on topics in the facilitator guide. At the conclusion of the session, all participants completed an evaluation/questionnaire (Appendix C) consisting of seven questions rated on a 5-point Likert scale (*Strongly agree/Agree/Neutral/Disagree/Strongly disagree*) and three open-ended questions:

1. How has today's session influenced your views and perspectives on global ophthalmology?
2. After today's session, what is one thing you may change in how you practice ophthalmology during residency?
3. After today's session, what is one thing you may change in how you practice ophthalmology in your future career?

We compiled and reviewed the answers to these three questions and categorized them by topic.

While all four iterations of the case study were facilitated by one of the authors (Michael C. Chen) familiar with the content of the case study and the discussion questions, we realize that subsequent facilitators will have varying degrees of expertise in the areas of global health, ophthalmology, and small-group facilitation. While expertise in these areas would be helpful, it is not absolutely necessary, as many of the learners likely will have had previous international, cross-cultural, and cross-socioeconomic experiences and the role of the facilitator is to guide the discussion to optimize peer learning. The facilitator's version of the case study (Appendix A) includes facilitator notes, and it is recommended that facilitators, prior to the session, read through and familiarize themselves with this document as well as with the limitations as described in the Discussion section below. Facilitators are also encouraged to review the publication "Facilitating Small Group Learning in the Health Professions," by Burgess, van Diggele, Roberts, and Mellis.[20]

Results

All participants ($N = 30$) were invited to fill out an evaluation/questionnaire after the session; the response rate was 100%. Answers to Likert-scale questions are displayed in the Figure. The activity was well received, with 100% of learners either strongly agreeing (90%) or agreeing (10%) when asked if the session was worthwhile and strongly agreeing (87%) or agreeing (13%) when asked if the format was conducive to meeting the learning objectives. Representative quotes from answers to the three open-ended questions are displayed in the Table. Answers to questions on how the session influenced learners' views and perspectives on global ophthalmology and how they would change how they practice ophthalmology in their residency and in their future careers revolved around the following topics: consideration of other perspectives, humility, self- and situational awareness, complexities of partnerships, reciprocity and exchange, importance of communication, and connection of principles between international and domestic medical practice.

Discussion

To address the need for more training resources to bring about greater self- and situational awareness of the complex relational, motivational, ethical, and logistical issues that may arise in international partnerships, we developed this case study to invite learners into a plausible real-world scenario and contemplate how these partnerships may be perceived differently by different

Copyright © 2024 Chen et al. This is an open-access publication distributed under the terms of the Creative Commons Attribution license.

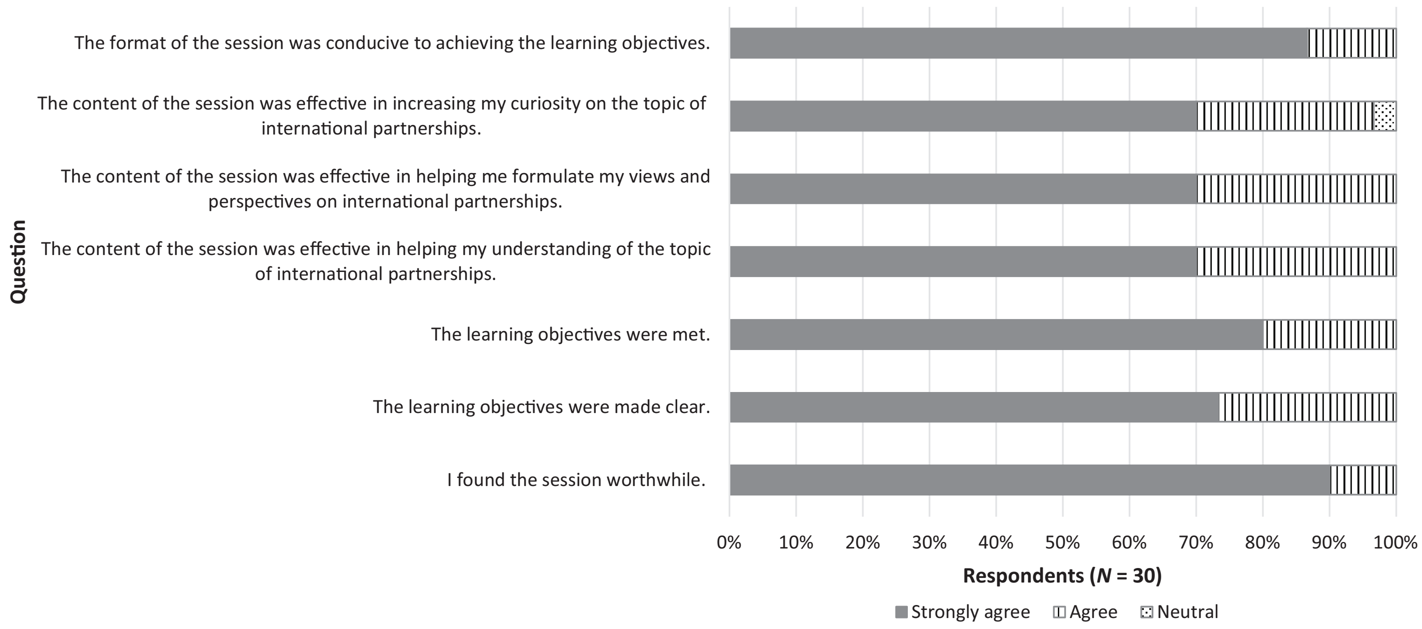

Figure. Learner responses to evaluation questions. Answers were rated on a 5-point Likert scale (*Strongly agree, Agree, Neutral, Disagree, Strongly disagree*).

stakeholders. By viewing the scenario through the eyes of four characters, learners explored the potential benefits of global health experiences and partnerships while at the same time considering how there may be both beneficial and detrimental effects on the various parties involved. We were pleased with the favorable reception of this case study activity by the learners—there was unanimous agreement that the activity was worthwhile and that the format was conducive to achieving the learning objectives. The evaluation/questionnaire comments indicated that the activity reached level 2 of Kirkpatrick's hierarchy of outcomes (change in knowledge, attitudes, and skills).[19]

In this case study, where the involved parties have motivations and goals that may or may not align with the other parties, learners are challenged to explore what is meant by the term *partnership,* as well as the healthy and unhealthy aspects of potential partnerships. Amidst the complexities, nuances, and trade-offs presented in this case study where the characters have noble aspirations but are also motivated by multiple other interests and may be bound by constraints outside of their control, learners are encouraged to collectively brainstorm aspects of a healthy partnership and discover that it is not simply one in which each stakeholder gets what they want out of the relationship but one in which there are collective goals, shared values, common purpose, and relational commitments that every stakeholder can aspire to.

Eichbaum and colleagues have brought attention to rethinking international partnerships and approaches and decolonizing global health education through transformative learning by way of a shifting of entrenched positions and assumptions and a challenging of preconceived notions.[21] We hope that our case study brings about this type of transformation as learners critically reflect on their own perspectives and compare and contrast them with the perspectives of the different characters in the scenario. As Guiles, Nuwagira, and Stone point out, while there are numerous global health educational activities to orient trainees before going abroad, humble and sensitive engagement with local colleagues and patients depends on the trainees' underlying assumptions about themselves and their hosts, which often may not manifest until trainees are on the ground participating in the global health experience.[22] As reflected in some of the learner comments, this case study was effective in calling out some of those assumptions in the classroom setting. By inviting learners to step into a narrative about a global health experience and to reflect on how they would think or would be viewed if they were each of the characters, they may be able to recognize and reflect critically on their assumptions prior to embarking on the trip or entering a partnership and to enter the situation more open to perspectives different from their own.

Due to scheduling and logistical constraints in running the sessions, group sizes were variable, with the smallest and largest group sizes being four and 15 learners, respectively. Although

Copyright © 2024 Chen et al. This is an open-access publication distributed under the terms of the Creative Commons Attribution license.

Table. Results of Thematic Content Analysis and Representative Quotes

Topic	Quotes
Consideration of other perspectives	"It was so great to hear the different perspectives of all the different key participants that are in some way involved or impacted by participation in global ophthalmology. The session forces you to take a step back and think about goals and motives of everyone involved."<br>"It's helpful to put ourselves in different shoes and think about how this partnership can come across from all perspectives."<br>"This session forces you to think about all the different perspectives involved. It allows you to take a step back and think about how small decisions, even well intended, have an effect on individuals from host countries."
Humility	"I'll try and practice with grace and the perspective that I don't know everything."<br>"Having a deeper understanding of my own limitations and not being too enthusiastic to work with or operate on patients when I know I might not be ready."<br>"Understand that there is learning to be done by everyone in the partnership."
Self- and situational awareness	"Before going into an international site I would want to take the time to ask what their needs/interests are and what would be a non-disruptive way to be involved."<br>"Helped me to think about how we are perceived in other countries."<br>"It gave me a perspective such that if I engage in [ophthalmology] globally I'll think a lot about how I leave my trace and how I can best help without taking away from the community."
Complexities of partnerships	"I have gained insight into the potential complexity of global partnerships, what defines a 'good' partnership, and the potential pitfalls that should be considered BEFORE a new partnership starts (and then considered frequently throughout its life)."
Reciprocity and exchange	"I think that my biggest takeaway from this case study is the shift in my view of global ophthalmology (and medicine, in general) from that of a charity to an opportunity to create partnerships. By sharing resources and skills available to me, I may be able to learn from my partners' expertise in practicing in lower resource settings, managing populations different from my own, or using techniques not available in my training, to improve care for all of our patients."<br>"This session has encouraged me to ensure that I stay engaged with colleagues from all parts of the world."
Importance of communication	"The session has expanded my views on this topic and urged me to consider the transactional nature of these relationships that are sometimes at play. I now recognize that these partnerships are better executed when clear expectations are set and communicated before a partnership is formed."<br>"This exercise made me realize how much open communication between providers and shared goals/objectives can impact delivery of care."<br>"I will be more conscious of the power of asking questions in my future career, and the importance of setting goals and objectives for professional partnerships early. I will be more conscious of revisiting these goals and objectives with future community partners often."
Connection of principles between international and domestic medical practice	"Our thoughts on global ophthalmology should include patients who do not have access to care close to home as well."<br>"I will try to keep an open mind when encountering patients from different cultures and not make assumptions based on cultural differences."<br>"I hope to have more open conversations with my colleagues about their ideas to improve our delivery of care to our patients, particularly to our underserved locally."

a group of four learners worked well for that particular session, as all four were engaged, we found that the session with 15 learners was difficult to facilitate, as this number did not allow sufficient opportunity for all participants to engage and share with the entire group and, at several points, unintended side conversations developed. While group dynamics may vary depending on the particular facilitator and learners, we agree with what has been reported in the literature, namely, that the ideal group size is likely five to eight learners per session.[20]

As the duration of this activity is 3 hours, toward the third hour focus and attention among the groups became more challenging to maintain. Because the text of the fourth part is particularly lengthy, a break between the third and fourth parts, as well as silent reading of the fourth part, can be considered.

The evaluation/questionnaire that was used at the end of the sessions asked if the learning objectives had been met and if the format of the session was conducive to achieving the learning objectives. Adding questions that probe more specifically about how the learning objectives have been met can be considered in future evaluations/questionnaires.

While this case study is directed at ophthalmology residents and medical students interested in global ophthalmology, the principles and themes presented in it are not exclusive to ophthalmology but may be applicable to other fields or situations in which international partnerships arise. Also, the themes and principles elicited in the case study are not exclusive to practicing medicine internationally. Given the diversity of the US patient population, the increasingly apparent resource limitations of the US health care system, and the need for multidisciplinary collaboration and partnerships, the case study's themes and principles may also be applicable to practicing medicine in the US. We envision that the case study could be included in pretrip preparation material not only for ophthalmology residency

Copyright © 2024 Chen et al. This is an open-access publication distributed under the terms of the Creative Commons Attribution license.

programs but also for other graduate and undergraduate medical education programs. It is our hope that the case study stimulates discussion among those involved with global ophthalmology and global health and encourages the development of other global health case studies, to encourage healthier partnerships and better informed medical work wherever the location may be.

Appendices

A. Case Study - Facilitator Version.docx

B. Case Study - Learner Version.docx

C. Session Evaluation Questionnaire.docx

All appendices are peer reviewed as integral parts of the Original Publication.

Michael C. Chen, MD: Associate Professor, Department of Ophthalmology, University of Colorado School of Medicine; Ophthalmology Division Chief, Denver Health Medical Center; ORCID: https://orcid.org/0000-0001-5178-4632

Judy Ndiritu, MBBS: Consultant Ophthalmologist and Vitreoretinal and Uveitis Specialist, Nyeri County Referral Hospital

Subash Bhatta, MD: Teaching Ophthalmologist and Vitreoretinal Consultant, Pacific Eye Institute

Disclosures
None to report.

Funding/Support
None to report.

Ethical Approval
Reported as not applicable.

Disclaimer
All names, characters, locations, and events portrayed in this case study are fictitious. Any resemblance to actual events, locations, or persons is entirely coincidental.

References

1. Bills CB, Ahn J. Global health and graduate medical education: a systematic review of the literature. *J Grad Med Educ*. 2016;8(5):685-691. https://doi.org/10.4300/JGME-D-15-00774.1
2. Peluso MJ, Forrestel AK, Hafler JP, Rohrbaugh RM. Structured global health programs in U.S. medical schools: a web-based review of certificates, tracks, and concentrations. *Acad Med*. 2013;88(1):124-130. https://doi.org/10.1097/ACM.0b013e3182765768
3. Camacci ML, Quillen DA, Montijo M, Chen MC. Applicants' interest in international ophthalmology during residency training: influence in selecting U.S. residency programs. *J Acad Ophthalmol (2017)*. 2018;10(1):e48-e54. https://doi.org/10.1055/s-0038-1641610
4. Camacci ML, Cayton TE, Chen MC. International experiences during United States ophthalmology residency training: current structure of international experiences and perspectives of faculty mentors at United States training institutions. *PLoS One*. 2019;14(11):e0225627. https://doi.org/10.1371/journal.pone.0225627
5. Sharfi D, Finklea B, Ehrlich JR, et al; American Academy of Ophthalmology Task Force on Academic Global Ophthalmology. Global ophthalmology fellowships: then and now. *Int Ophthalmol Clin*. 2023;63(1):33-38. https://doi.org/10.1097/IIO.0000000000000449
6. Cole E, Mathenge W, Kiage D, et al; American Academy of Ophthalmology Task Force on Academic Global Ophthalmology. GO-ing for gold: global perspectives on creating a gold standard for global ophthalmology (GO) education. *Int Ophthalmol Clin*. 2023;63(1):5-13. https://doi.org/10.1097/IIO.0000000000000446
7. Lu PM, Park EE, Rabin TL, et al. Impact of global health electives on US medical residents: a systematic review. *Ann Glob Health*. 2018;84(4):692-703. https://doi.org/10.29024/aogh.2379
8. Lu PM, Mansour R, Qiu MK, Biraro IA, Rabin TL. Low- and middle-income country host perceptions of short-term experiences in global health: a systematic review. *Acad Med*. 2021;96(3):460-469. https://doi.org/10.1097/ACM.0000000000003867
9. White M, Evert J. Developing ethical awareness in global health: four cases for medical educators. *Dev World Bioeth*. 2014;14(3):111-116. https://doi.org/10.1111/dewb.12000
10. Asao S, Lewis B, Harrison JD, et al. Ethics simulation in global health training (ESIGHT). *MedEdPORTAL*. 2017;13:10590. https://doi.org/10.15766/mep_2374-8265.10590
11. Elansary M, Peluso M, Kallem S, Thomas J, Rabin T. Global health clinical ethics. *MedEdPORTAL*. 2015;11:10232. https://doi.org/10.15766/mep_2374-8265.10232
12. Fredrick NB. Exploring health systems within the context of social determinants of health: a global health case study. *MedEdPORTAL*. 2016;12:10457. https://doi.org/10.15766/mep_2374-8265.10457
13. Fallah PN, Ovsak GG, Kasper J, et al. A longitudinal case-based global health curriculum for the medical student clerkship year. *MedEdPORTAL*. 2020;16:11038. https://doi.org/10.15766/mep_2374-8265.11038
14. Lawrence Z, Szyld D, Williams R. Diarrhea in the returning traveler: a simulation case for medical students to learn about

Copyright © 2024 Chen et al. This is an open-access publication distributed under the terms of the Creative Commons Attribution license.

global health. *MedEdPORTAL*. 2020;16:10935. https://doi.org/10.15766/mep_2374-8265.10935

15. McLean SF. Case-based learning and its application in medical and health-care fields: a review of worldwide literature. *J Med Educ Curric Dev*. 2016;3:S20377. https://doi.org/10.4137/JMECD.S20377
16. Nohria N. What the case study method really teaches. *Harvard Business Review*. December 21, 2021. Accessed July 9, 2024. https://hbr.org/2021/12/what-the-case-study-method-really-teaches
17. Educational activity—global ophthalmology. American Academy of Ophthalmology. February 26, 2020. Accessed July 9, 2024. https://www.aao.org/education/ethics-detail/educational-activity–global-ophthalmology
18. Thomas PA, Kern DE, Hughes MT, Chen BY, eds. *Curriculum Development for Medical Education: A Six-Step Approach*. 3rd ed. Johns Hopkins University Press; 2016.
19. Kirkpatrick DL. *Evaluating Training Programs: The Four Levels*. *Berrett-Koehler*; 1994.
20. Burgess A, van Diggele C, Roberts C, Mellis C. Facilitating small group learning in the health professions. *BMC Med Educ*. 2020; 20(suppl 2):457. https://doi.org/10.1186/s12909-020-02282-3
21. Eichbaum QG, Adams LV, Evert J, Ho MJ, Semali IA, van Schalkwyk SC. Decolonizing global health education: rethinking institutional partnerships and approaches. *Acad Med*. 2021;96(3): 329-335. https://doi.org/10.1097/ACM.0000000000003473
22. Guiles DA, Nuwagira E, Stone GS. Four learner categories in global health experiences: a framework for successful resident engagement. *Ann Glob Health*. 2022;88(1):66. https://doi.org/10.5334/aogh.3562

Received: October 3, 2023
Accepted: April 10, 2024
Published: August 9, 2024

Copyright © 2024 Chen et al. This is an open-access publication distributed under the terms of the Creative Commons Attribution license.
